# Improved Distorted Configuration Space Path Planning and Its Application to Robot Manipulators

**DOI:** 10.3390/s20216060

**Published:** 2020-10-24

**Authors:** Yangmin Xie, Rui Zhou, Yusheng Yang

**Affiliations:** Shanghai Key Laboratory of Intelligent Manufacturing and Robotics, School of Mechatronics Engineering and Automation, Shanghai University, Shanghai 200444, China; xieym@shu.edu.cn (Y.X.); ruizhou@shu.edu.cn (R.Z.)

**Keywords:** path planning, obstacle avoidance, robot manipulator, distorted configuration space

## Abstract

Real-time obstacle avoidance path planning is critically important for a robot when it operates in a crowded or cluttered workspace. At the same time, the computational cost is a big concern once the degree of freedom (DOF) of a robot is high. A novel path planning strategy, the distorted configuration space (DC-space) method, was proposed and proven to outperform the traditional search-based methods in terms of computational efficiency. However, the original DC-space method did not sufficiently consider the demands on automatic planning, convex space preservation, and path optimization, which makes it not practical when applied to the path planning for robot manipulators. The treatments for the problems mentioned above are proposed in this paper, and their applicability is examined on a three DOFs robot. The experiments demonstrate the effectiveness of the proposed improved distorted configuration space (IDCS) method on rapidly finding an obstacle-free path. Besides, the optimized IDCS method is presented to shorten the generated path. The performance of the above algorithms is compared with the classic Rapidly-exploring Random Tree (RRT) searching method in terms of their computation time and path length.

## 1. Introduction

Path planning is to find a route for the robots from the start position to the target position without colliding with obstacles [1,2]. Since many robots work in crowded and cluttered environments, the obstacle avoidance problem, in general, is not straightforward to solve [3]. Most of the traditional path planning algorithms can be classified into several categories: graph search methods [4], sampling-based method [5], and intelligent bionic methods [6]. The most widely used graph search algorithm is the A* search method [7], which was developed by Hart in 1968. It has a heuristic algorithm to lead the search toward the goal, and the designed heuristic function has a significant effect on the performance of A* search [8]. The computational cost and data storage expand exponentially to the length of the path, which induces the low calculation speed, especially in high-dimensional spaces. The sampling-based methods [9,10], such as random-exploring rapid tree (RRT) algorithms [11] and probabilistic roadmap method [12], are randomized approaches with merits in providing a fast solution in high-dimensional maps and adopted as the path planning solution in many research works for robot manipulators [13]. However, when there are a lot of obstacles or narrow channels in the working environment, the convergence speed of the algorithm is slow, and the efficiency is considerably reduced [14]. At the same time, due to the nature of randomness, the planned paths can tremendously vary from time to time [15]. Commonly intelligent bionic algorithms include ant colony optimization [16,17] and genetic algorithm [18,19], among others. They have strong robustness but also the limitation of being computationally intensive [20,21].

Compared with the above methods, path planning in the DC-space is a new concept presented in [22]. With the innovative idea to diminish the obstacles in a distorted space, the algorithm successfully abandons the tedious process to find an obstacle-free path. Instead, a straight line connecting the start and target points in the DC-space fulfills the purpose, which is distinctive to any path planning method mentioned above. Additionally, the DC-space method has been proven to be much more efficient than A* and PRM methods [22].

However, we found that the original DC-space method left some problems unsolved, which could lead to failures when it is applied to real robot manipulators. The first problem is that the collapse points, where all the obstacles are distorted to in the DC-space, are hand-picked by users. This requirement does not only slow down the process but also could lead to improper choices of the collapse points and thus, planning failures. The second problem is that the convex property of the DC-space is not preserved when the occupied cells attach with the boundary surfaces. The violation of the convex space assumption can make the straight line connecting the start and target points pass through the invalid area, which does not provide a feasible path in the original configuration space. The third problem, as it was stated in [22], is the resulted solution should be post-processed to provide an optimized path with fewer detours.

Targeting at solving the aforementioned problems in the original DC-space method, the work in this paper presents solutions one by one. Besides, the algorithm to generate maps in the configuration space is also introduced to facilitate its application on robot manipulators. The major contributions include

(1) An automatic collapse point generation algorithm is proposed to calculate the locations where the obstacles are distorted, which not only provides an autonomous procedure for the DC-space method but also avoids topology destruction due to bad choices of the collapse points.
(2) Boundary preservation is achieved using a node growth algorithm, which ensures the DC-space to be convex no matter where the obstacles locate.
(3) An optimizing algorithm is used to post-process the path, which shortens the original path to a considerable extent.
(4) The standard procedure to implement the improved DC-space method above is presented and demonstrated on a three DOF robot.

The following sections are arranged as follows: The configuration space generation method based on the task space is introduced in Section 2 first. Then, Section 3 reviews the conventional principles of path planning in DC-space and presents its problems, which serve as the motivation of this paper. Next, the theory of the IDC-space method is proposed in Section 4 to address the problems, and the efficiency and performance of the proposed IDC-space method are discussed in simulation environments in Section 5. The experiments with one real robot manipulator are illustrated in Section 6 to demonstrate the implementation and effectiveness of the IDC-space method. Finally, Section 7 concludes the paper.

## 2. Paper Framework

**Figure 1:**
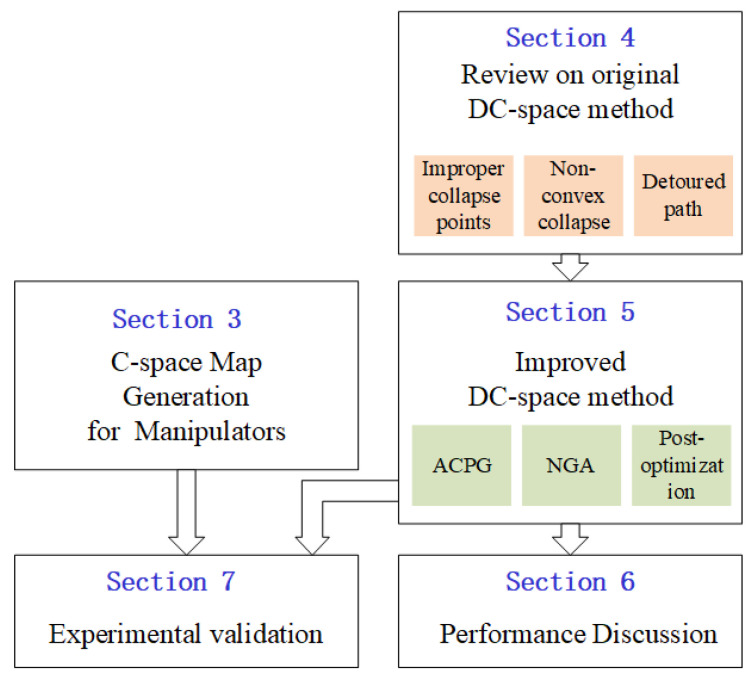
Framework of the contents.

In this section, the outline of this paper is presented for the readers to have a clear technical path of the contents in this paper. The overall framework is shown in Figure 1. The work in this paper follows the assumptions listed below. They fit most of the industrial application scenarios where manipulators are designed to work in monitored environments and streamlining fashion:

(1) The robot manipulator is fixed in the workspace;
(2) The environment is static;
(3) The kinematics of the manipulator and the position of the obstacles are known as a priori;
(4) The boundaries of the configuration space, corresponding to the joint motion limits of the manipulator, are pre-known and pre-defined to form a close convex space.

To apply the DC-space method on a real robotic system, the mission in task space needs to be converted to a path planning problem in the configuration space. A systematic way for this conversation is introduced in Section 3 as a preliminary module for path planning. Once a robot manipulator is mounted, its kinematic relationship with the workspace is fixed and treated as a priori information for the map generation.

Section 4 mainly reviews the original DC-space method, and more importantly, lists the possible failures it could encounter when applied to a real system. The three theoretical problems, improper collapse points, non-convex collapse and detoured path, are discussed using simple cases.

Targeting at solving the problems, corresponding solutions are provided in Section 5, which is the main innovation of this paper. (1) The automatic collapse point generation (ACPG) algorithm collapses the obstacles in the configuration space automatically by minimizing the total potential energy of the system. (2) The node growing algorithm (NGA) is proposed to ensure the boundary obstacles shrink onto the boundary surface, and thus the convex property of the distorted space is preserved. (3) Based on the collision-free principle, a trajectory optimization algorithm is proposed to make short cuts of the original path generated by the DC-space method.

We analyze and examine the performance with the aforementioned improvements in Section 6. The comparisons between the improved DC-space method and one widely used path planning algorithm, the rapidly exploring random tree method, are conducted to show its high computational efficiency. The influences of the map conditions and size on the computation time are discussed, and the path length and time cost with and without post-optimization are compared. Applying the map generation method in Section 3 and the improved DC-space method in Section 5, an experiment on a manipulator is conducted to demonstrate the effectiveness of the proposed method on real systems, and the results are discussed in Section 7.

## 3. Map Generation in C-Space

The configuration space is the fundamental concept used in many robot motion planning applications [23,24]. Different from the physical workspace, the dimensions of the C-space are defined as the control variables of all the DOFs in the robotic system [25]. The first task to apply any algorithm in C-space for obstacle-free path planning is to transform the observable obstacle map in the task space to the C-space [26]. We present the process in this section to illustrate a systematic way to accomplish the task.

For an articulated robot *R*, its configuration c can be described as a *d*-dimensional vector that specify the state of each DOF. Convex configuration space for a specific robot is confined with upper boundaries $\left\{{\bar{c}}_{0},{\bar{c}}_{1},\cdots ,{\bar{c}}_{d-1}\right\}$ and lower boundaries $\left\{{\underline{c}}_{0},{\underline{c}}_{1},\cdots ,{\underline{c}}_{d-1}\right\}$. With certain discretization, the configuration space can be represented as $C=\left\{c_{g}|c_{g}={\left\{c_{0},c_{1},\cdots ,c_{d-1}\right\}}_{g},\;g=1,\;2,\;\cdots ,N_{g}\right\}$, where *d* is the number of the DOF of the robot, and $N_{g}$ is the number of all cells in the gridded C-space. Possible constraints from the mechanical system of the robot can induce unreachable areas in C, and the cell set belongs to that area is denoted as $C_{ur}$. It naturally becomes unpassable occupied cells in C.

**Figure 2:**
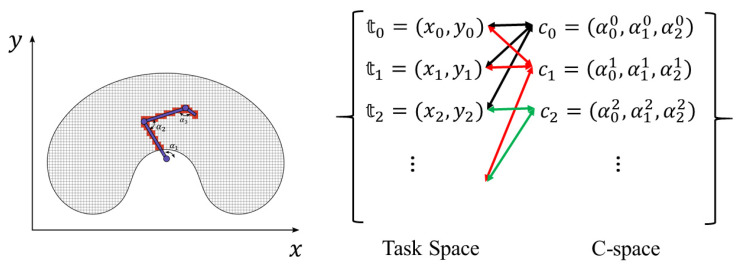
One example of a bidirectional projection between the task space and the configuration space, both of which are discretized. For each reachable configuration of the robot, its occupied cells (in red) in task space can be calculated and stored first.

Denote the set of the reachable cells in C as $C_{r}$. For all $c\in C_{r}$, denote the set of the cells in the discretized task space T occupied by the whole robot body as $T_{oc}\left(c\right)$, which can be calculated with the kinematic model of the robot. Calculate $T_{oc}\left(c\right)$ for all the elements in $C_{r}$ and a bidirectional projection can be established between T and $C_{r}$, as shown in Figure 2. For a specific robot, this projection relationship is only dependent on its kinematics and is invariant, so it can be pre-calculated and stored for further usage.

When the obstacles in the task space are detected, the set of the occupied cells $T_{ob}$ in the task space can be obtained. For all the $𝕥\in T_{ob}$, find their projections in $C_{r}$ and the union of all the projections $C_{ob}$ becomes the obstacle projections in C. The non-passable cells in C are the union of the $C_{ob}$ and $C_{ur}$, which generates an ‘obstacle’ map in C-space. Generally, the original obstacle in the task space is expanded in the first place to guarantee a safe distance between the planned path and the obstacles.

For a particular robot in a workspace, different situations of obstacles correspond to distinct maps in the C-space. However, once the robot is mounted and the work environment is static, the C-space is fixed, as well as the corresponding DC-space. In this case, the DC-space can be calculated ahead of time and stored offline before path planning, so that it reduces the burden of computation and improves the efficiency of path generation.

## 4. DC-Space Method Review and Discussion

Path planning in the DC-space, where all obstacles are collapsed into low dimension geometric objects, can find a feasible path quickly. A brief review of the DC-space method by Chen [22] is presented in Section 4.1. However, several problems were not properly addressed in the very first version of the DC-space method, which will be illustrated in Section 4.2 and serve as the motivations for the work in this paper.

### 4.1. Conventional DC-Space Path Planning Method

**Figure 3:**
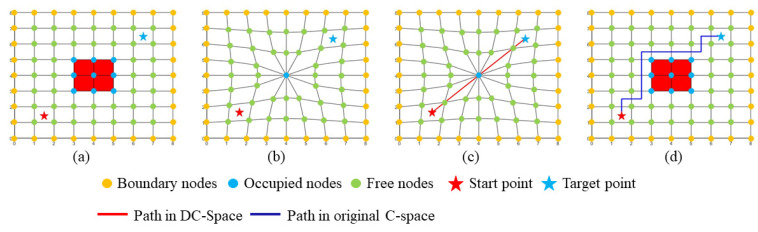
The process of path planning in DC-space. (**a**) C-space map. Grid nodes can be categorized into three types, boundary nodes (orange), free nodes (green), occupied nodes (blue). Occupied cells are colored in red, the start point (red star), and the target point (blue star) locate at the geometric center of the start cell and the target cell separately. (**b**) Obstacles collapse. The blue point is the collapse position for all the nodes in one obstacle. The free nodes are displaced accordingly. (**c**) Path generation in the DC-space. The start point and the target point are connected with one straight path (in red) directly. (**d**) Path restoration in the C-space. A feasible path (dark blue) that avoids the obstacle is built after projecting the straight path in DC-space back to the C-space.

The process of the conventional DC-space path planning method includes four steps: (1) C-space map initialization, (2) obstacles collapse, (3) path generation in DC-space, (4) path restoration in C-space, as shown in Figure 3.

In the C-space map initialization step, the obstacles in the task space are transformed into the configuration space, as presented in Section 3. The configuration space $C\;\epsilon \;R^{n}$ is formed as a convex space, where n is the DOF of the robot. The occupied obstacle cells can be clustered as $T=\left\{T_{i}|i=1,2,\cdots ,n_{t}\right\}$, where $T_{i}$ is the *i*th cluster of the obstacle cells and $n_{t}$ is the total number of the obstacle clusters. Denote the grid nodes for $T_{i}$ as $N_{occ}^{i}$, the boundary nodes of the C-space as $N_{boun}$, and the rest nodes as free nodes $N_{free}$. Then the overall grid nodes N can be represented by $N=\left(\sum_{i=1}^{n_{t}}N_{occ}^{i}\cup N_{boun}\cup N_{free}\right)$. The coordinate of the grid node is an n-dimension vector defined as $v_{g}={\left(x_{i^{1}\ldots i^{n}}^{1},\cdots ,x_{i^{1}\ldots i^{n}}^{j},\cdots x_{i^{1}\ldots i^{n}}^{n}\right)}_{g}^{T}$, where $x_{i^{1}\ldots i^{n}}^{j}$ is the coordinate along *j*th axis and $i^{k}$ is the cell index in the direction of the *k*th axis.

The obstacle collapse step is the core of the DC-space method, where each obstacle $T_{i}$ is compressed into the object $P_{i}^{T}$ in a subSpace $R^{m}$, where *m* is less than *n* − *1*. Obstacle collapsing leads to the position offsets of $N_{free}$, as shown in Figure 3b, which generates a distorted space. The coordinate of each node in $N_{free}$ after distortion is denoted as $vd_{g}={\left(y_{i^{1}\ldots i^{n}}^{1},\cdots ,y_{i^{1}\ldots i^{n}}^{j},\cdots y_{i^{1}\ldots i^{n}}^{n}\right)}_{g}^{T}$ and can be calculated by Theorem 1 [22]:

**Theorem** **1.** *In a stable distorted grid, a free node sits at the geometric center of its neighboring nodes. Thus,* $vd_{g}$ *can be calculated by the following formula*

(1) $$y_{i^{1}\ldots i^{n}}^{j}=\frac{1}{2n}\left(y_{\left(i^{1}-1\right)i^{2}\ldots i^{n}}^{j}+y_{\left(i^{1}+1\right)i^{2}\ldots i^{n}}^{j}+y_{i^{1}\left(i^{2}-1\right)\ldots i^{n}}^{j}+y_{i^{1}\left(i^{2}+1\right)\ldots i^{n}}^{j}+\ldots +y_{i^{1}i^{2}\ldots \left(i^{n}-1\right)}^{j}+y_{i^{1}i^{2}\ldots \left(i^{n}+1\right)}^{j}\right)$$

*where* j=1,⋯,n*. The Equation (1) indicates that the coordinate of* $y_{i^{1}\ldots i^{n}}^{j}$ *in the* jth *axis is the average of the coordinates of all adjacent nodes in the same dimension.*

The path generation is straight-forward in the DC-space since all the obstacles are collapsed into the lower dimension space and basically “disappear” in the high dimension space. With the given start point p and the target point q, the path $L^{\prime }$ in DC-space is generated by connecting them with one straight line directly.

Finally, in order to project the path $L^{\prime }$ back to the path L in the C-space, corresponding cells for the path L cell indexes are connected in the same sequence as the path $L^{\prime }$, which generates a final feasible obstacle-free path. More details about the original version of the path planning in DC-space can be found in [22].

### 4.2. Infeasibility Case Analysis

Path planning in DC-space is an efficient algorithm to tackle the path planning problem and is particularly effective in decreasing the calculation cost when the dimension of the configuration space is high. However, when examining the implementation of the original DC-space method on real robotic systems, some neglects of the obstacle properties could lead to failures of the method.

#### 4.2.1. Improper Choice of the Collapse Point

**Figure 4:**
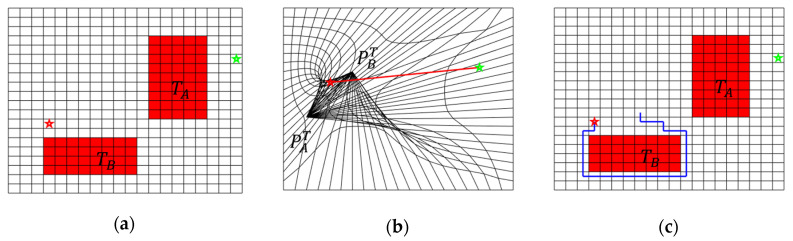
One 2D example of path planning failure in DC-space when improper distortion points are chosen for obstacles. (**a**) Initial C-space with obstacles ($T_{A}$ and $T_{B}$), the start point (red star), and the target point (green star). (**b**) The DC-space after the obstacles collapse, the upper-right obstacle $T_{A}$ is distorted to the lower-left position $P_{A}^{T}$, and the lower-left obstacle $T_{B}$ is distorted to the upper-right position $P_{B}^{T}$. Penetration between grids is observed. (**c**) The generated path (blue) in C-space falls into infinite loops and cannot achieve the target.

The position of the collapse points for the occupied cells has a significant influence on the final DC-space. However, they were chosen manually, which could lead to unpredictable distortion and penetration among cells. Figure 4 illustrates one example of the path planning in DC-space, where the collapse points for the obstacles are specified improperly. The upper right obstacle is twisted to the lower left position, and the lower left obstacle is twisted to the upper right position, which results in the intersection and overlap of the grid in the middle region of two distortion points. The start point is distorted to the overlapping area due to the deformation. Under such a circumstance, even though one straight path is built between the start point and the target point in the DC-space, the path falls into an infinite loop when mapping back to the original C-space, and no effective path can be found, as shown in Figure 4c. To prevent the destruction of the continuous and smooth topology in DC-space, this paper presents an automatic collapse point generation algorithm that can preserve the topology of the original C-space, which will be illustrated in Section 5.1.

#### 4.2.2. Non-Convex DC-Space

**Figure 5:**
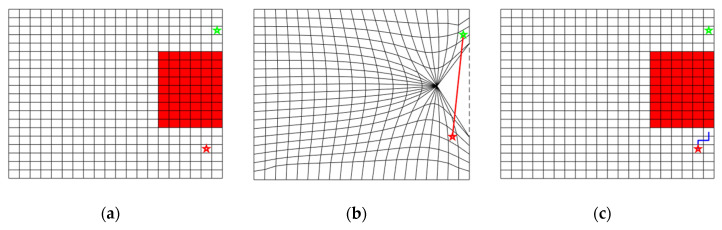
Non-convex DC-space situation. (**a**) The obstacle is connected with one boundary of C-space. (**b**) The generated DC-space after deforming the obstacle, and one path (red line) connects the start point (red star) and the target point (green). (**c**) The path is generated in C-space after mapping back from DC-space. The path stops extending when reaching the boundary.

Figure 5a shows a case that part of the occupied nodes are also the boundary nodes, which is a common situation in practice. When the collapse point is not on the boundary, which is the case in the conventional DC-space method, the convex feature of the DC-space would be lost, as shown in Figure 5b. Without constraining the boundary nodes on the original boundaries, the deformed boundary would be collapsed into a non-convex shape. This violates the fundamental assumption in the DC-space method and could lead to path planning failure, as shown in Figure 5b,c. Even though the start and target points are both inside the boundaries, their straight-line connection could pass through the “vacuum area” where no valid configuration cells exist. Therefore, no meaningful path will be generated because some part of the path in the DC-space is beyond the original configuration space. The generated path has to stop extending when it researches the boundary.

In this paper, to preserve the convex structure of the boundary, the boundary nodes are restricted on the boundary surface. An automatic strategy is developed in Section 5.2 to determine how the nodes of a boundary-attached obstacle should be deformed into the corresponding boundary nodes.

#### 4.2.3. Detoured Path

**Figure 6:**
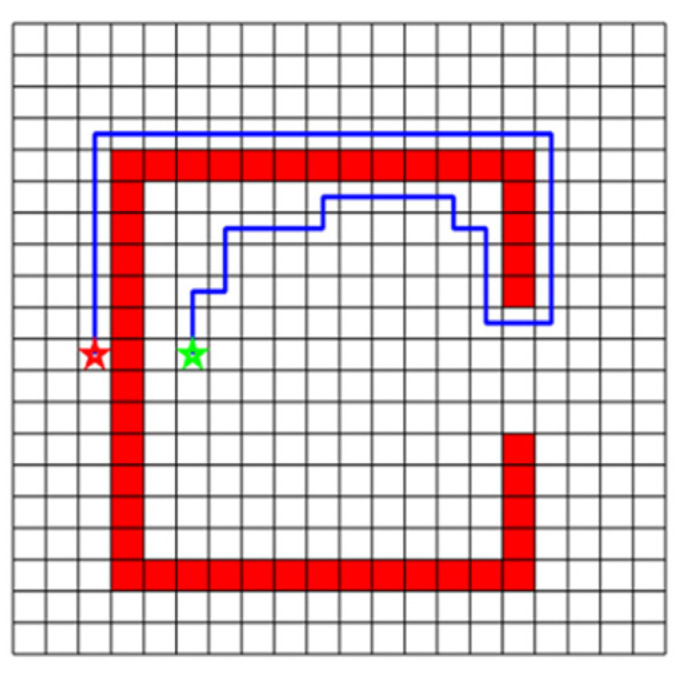
One example of the tortuous path by the original DC-space method. A red star denotes the start point, and a green star denotes the target point.

Connecting the start point p and target point q with the straight line is the most efficient strategy of path planning in DC-space. However, the mapped back trajectory in C-space tends to be tortuous, as shown in Figure 6. This is due to the tendency of the path in the distorted space to be attracted by the collapsed obstacle. The distortion of the space near the collapse point results in dense cell distribution in the local area, and the grid cells away from the collapse point are relatively sparse. Therefore, when the straight line passes through the dense area, it can cross a large number of cells, even only traveling by a small distance in the DC-space. The projected path in the C-space, as a result, tends to be close to the obstacles’ periphery and is largely detoured.

Aiming at obtaining a shorter path, a trajectory optimization algorithm is presented in Section 5.3 to reduce unnecessary path nodes, which connects distant path nodes and delete unnecessary ones based on the collision-free principal.

## 5. Improved DC-Space Path Planning

This section introduces several treatments to tackle the aforementioned problems, which are separately introduced in detail and demonstrated by examples in the following contents.

### 5.1. Automatic Collapse Point Generation

In the original DC-space method, the locations of the free nodes in the distorted space are automatically generated by minimizing the potential energy when springs with stiffness of K is assumed among the free nodes. However, the locations of the collapsed obstacles are manually assigned, which is not only troublesome but more damagingly could cause destruction of the topology in the distorted space if the collapse locations are not properly chosen.

In this paper, the algorithm is changed to let the collapsed obstacles floating in the distorted space, and their final ceased locations, the same as the free nodes, are determined by minimizing the total potential energy of the system. This helps the obstacles to settle down without being designated the locations and preserves the smooth space structure with pure elastic deformations, which is named as the Automatic Collapse Point Generation (ACPG) algorithm in this paper.

With the unified idea above, the distortion point $P_{i}^{T}$ for the floating obstacle $T_{i}$ would lie on the geometric center of its surrounding nodes. Let $S_{i}=\left\{S_{i}^{1},S_{i}^{2},\cdots ,S_{i}^{N_{s}^{i}}\right\}$ be the surrounding node set for the ith obstacle and these nodes belong to $N_{free}$ or $N_{boud}$, where $N_{s}^{i}$ is the number of the surrounding nodes, then $P_{i}^{T}$ follows the equation:

(2) $$P_{i}^{T}=\frac{1}{N_{s}^{i}}\left(vd_{S_{i}^{1}}+vd_{S_{i}^{2}}+\cdots +vd_{S_{i}^{N_{s}^{i}}}\right)$$

Nodes in $S_{i}$ belongs to $N_{boun}$ is treated as the surrounding boundary nodes of the obstacle, and those nodes are restricted to be fixed on the boundary to preserve the convex structure of the boundary in DC-space. Thus, the positions of nodes in $S_{i}$ satisfy:

(3) $$\forall s\in \left\{S_{i}|S_{i}=\left\{S_{i}^{1},S_{i}^{2},\cdots ,S_{i}^{N_{s}^{i}}\right\}\right\},vd_{s}=\{\begin{array}{c} vd_{s},\;\;if\;s\in N_{free}\\ v_{s},\;\;if\;s\in N_{boun} \end{array}$$

where $v_{s}$ and $vd_{s}$ are the positions of the surrounding node s before and after the distortion.

**Figure 7:**
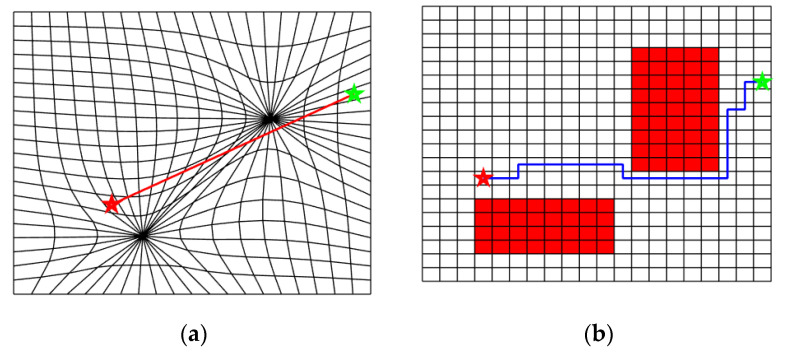
(**a**) The resulted DC-space for the situation in Figure 4. The start position and the target position are connected with one straight line directly. (**b**) The effective path after projecting the path back to C-space.

With the ACPG algorithm, the case in Figure 4 has a feasible path output, as shown in Figure 7. The autonomously generated distorted space has a much smooth structure, and the resulted path in C-space is much reasonable.

**Figure 8:**
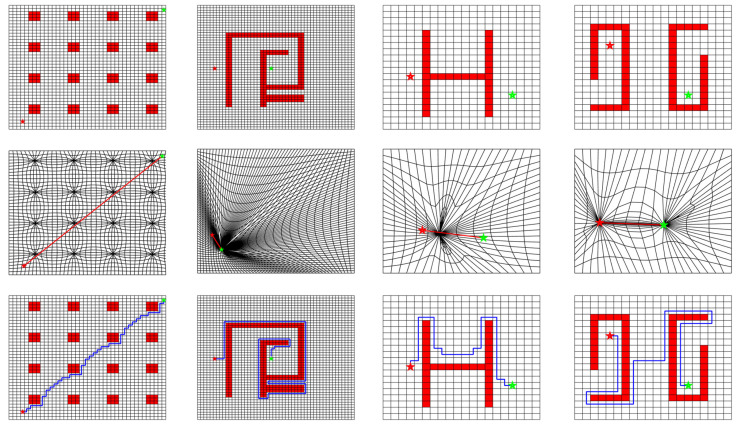
Some examples with floating obstacles. The first row is the initial C-space with obstacles. The second row presents the resulted DC-space after applying ACPG method. The start point (red star) and the target point (green star) are connected with one straight line directly. The third row is the generated path after projecting the path back to the C-space.

The ACPG method avoids the uncertainty of manually selecting of collapse points and helps to improve the efficiency and intelligence of the DC-space algorithm. The obstacle collapse is simultaneously achieved as computing the distortion positions for $N_{free}$, this maintains a smooth topology relationship among cells in the DC-space. More examples with complex obstacle distributions are presented in Figure 8 to verify the applicability and effectiveness of the proposed ACPG method.

### 5.2. Boundary Preservation

To deal with the non-convex problem in the original DC-space method, we add a strict constraint that the boundary nodes should stay unmoved in this improved DC-space method. For the floating obstacles lying inside the boundary, the collapse points can be computed with the ACPG method; however, when the obstacles attach with the boundary, the ACPG method will fail since the obstacles could not simply collapse into one single point anymore.

If an obstacle $T_{i}$ attaches with the boundary of the original C-space, then occupied nodes $N_{occ}^{i}$ belong to $T_{i}$ can be separated into two groups: nodes that are the intersection of $N_{occ}^{i}$ and $N_{boun}$ are denoted as $NB^{i}$ (Equation (4)), and the complementary set of $NB^{i}$ in $N_{occ}^{i}$ is denoted as $NP^{i}$ (Equation (5)). In order to maintain a convex boundary for this specific case, the nodes in $NB^{i}$ are restricted to be stationary in this paper, and the nodes in $NP^{i}$ will collapse into the boundary nodes in $NB^{i}$:

(4) $$NB^{i}=N_{occ}^{i}\cap N_{boun}$$

(5) $$NP^{i}=C_{N_{occ}^{i}}NB^{i}$$

**Figure 9:**
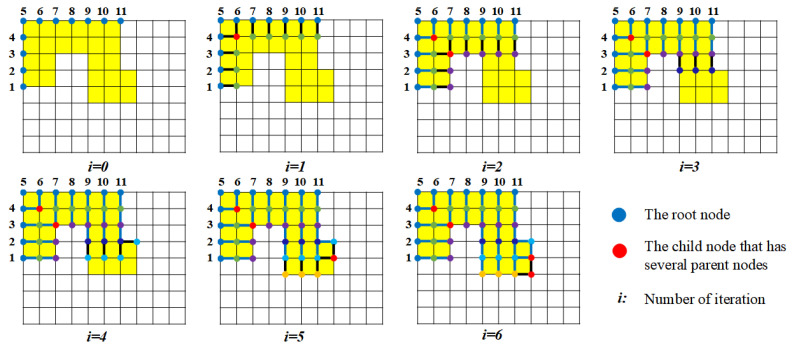
The process of iterations for growing from root nodes. The number in the picture is the index of the root node. The child nodes grow in the same iteration are in the same color.

The critical problem becomes how to determine the corresponding node in $NB^{i}$ for each node in $NP^{i}$ to achieve such a controlled collapse. A node growing algorithm (NGA) is proposed to solve the problem. The nodes in $NB^{i}$ are regarded as the root nodes, and the nodes in $NP^{i}$ are treated as the branch nodes that grow from the root nodes. In the growing process, each node in $NP^{i}$ is labeled by two indexes: one is the root index where this node grows from, and the other one is its direct parent node index. The process is shown in Figure 9. The root nodes serve as the first layer of the parent nodes. In each iteration of growth, the parent nodes of the current layer grow to their unassigned neighbor nodes, which will become their child nodes. If one child node is reached by multiple parent nodes, the parent indexes of this node are the combination of all. For the next round of growth, the child nodes become new parent nodes, and the growth process continues until every node in $NP^{i}$ is labeled. Eventually, the parent-child relationship between nodes in $NB^{i}$ and nodes in $NP^{i}$ can be generated, which inherently determine the corresponding relationship between the nodes in $NB^{i}$ and $NP^{i}$. The details of NGA are presented in Algorithm 1.
**Algorithm 1.** Node growing algorithm**Input:** C-space, $T_{i}$, $N_{occ}^{i}$, $NB^{i}$, $NP^{i}$**Output:** leaf nodes for each root node**Initialization:**
$iteration_{num}$ = 1; $Node_{parent}$ is empty.**for** each obstacle $T_{i}$ in $T=\left\{T_{i}|i=1,2,\cdots ,n_{t}\right\}$
**do** **if**
$T_{i}$ attaches with the boundary of C-space **do**  separate occupied nodes $N_{occ}^{i}$ into $NB^{i}$ and $NP^{i}$  **while** nodes in $NP^{i}$ are not searched totally **do**   **if**
$iteration_{num}=1$
**do**    root nodes $Node_{root}$ are nodes in $NB^{i}$, and each root node has one index as a root index    $Node_{parent}=Node_{root}$    The root index for a node in $Node_{parent}$ is its original root index   **end if**   **for** each node $g_{p}$ in $Node_{parent}$
**do**    searching one-ring neighboring nodes $Node_{child}$ from $NP^{i}$ in all possible directions    $g_{p}.append\left(Node_{child}\right)$
   **end for**   $Node_{parent\_temp}=\{\}$
   **for** each node $g_{p}$ in $Node_{parent}$
**do**    **for** each child node $g_{n}$ in $g_{p}$
**do**     **if**
$g_{n}$ is searched before **then**      stop searching in this direction     **else**
**if**
$g_{n}$ is searched by two or more parent nodes **then**      label $g_{n}$ by root indexes of its all parent nodes     **else**
**if**
$g_{n}$ is not searched before **then**      label $g_{n}$ by the root index of its parent node      $Node_{parent\_temp}.append\left(g_{n}\right)$     **end if**    **end for**   **end for**
   $iteration_{num}+=1$   $Node_{parent}=Node_{parent\_temp}$  **end while** **end if****end for**

**Figure 10:**
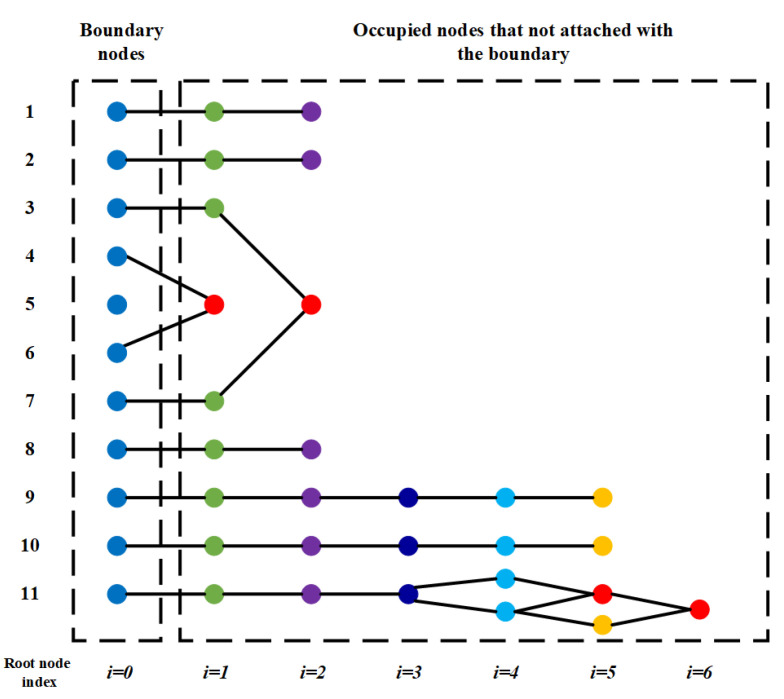
The hieratical relationship between the root nodes and the child nodes for the example in Figure 9. The child nodes generated from the same root node will be collapsed at that root node. The child node that has several parent nodes (in red) will be deformed into the middle position of the trajectory generated by its all parent nodes along the boundary.

The idea of NGA is to compress the nodes generated from the same root node to the position of this root node. With careful examination of the DC-space method, it is only the collapsed locations of the edge nodes that would influence the distorted space. If a node has only one parent index, the node will be compressed to the position of the root node that corresponds to the root index of the parent node. If the node has two or more parent indexes along the chain, the middle position on the boundary of all corresponding root nodes is taken as the distortion position for this node. Take the obstacle in Figure 9 as an example. The NGA results in the obstacle node relationship structured in Figure 10.

**Figure 11:**
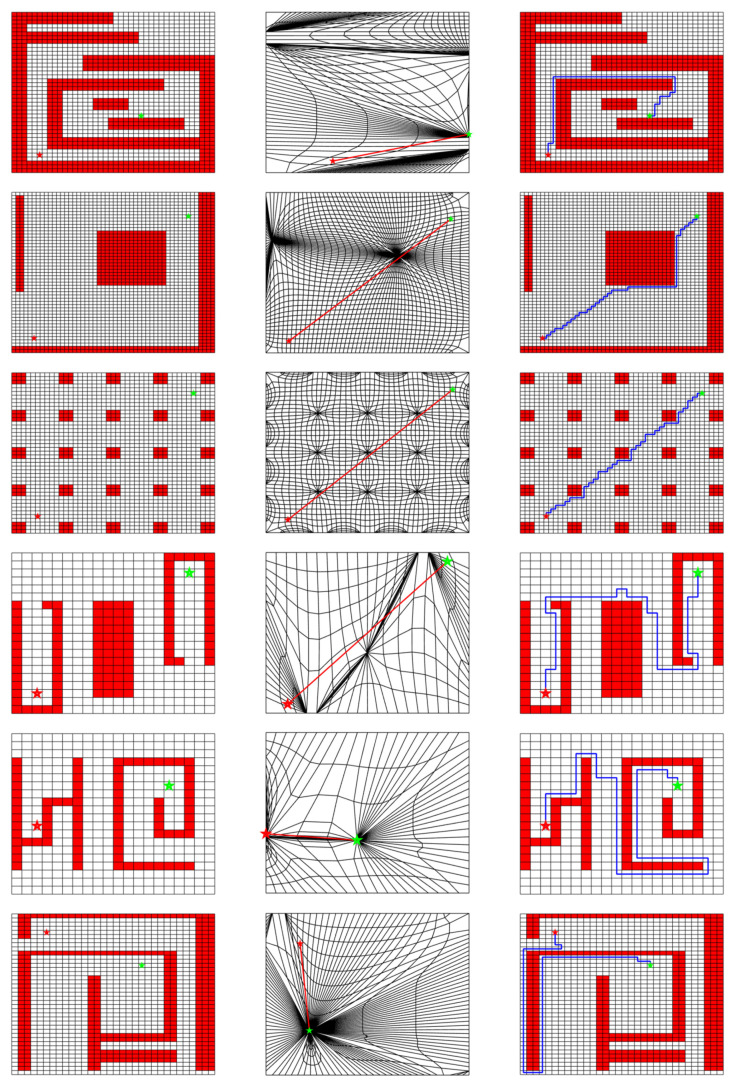
The examples of C-space with mixed types of obstacles. The first column is the initial C-space. The second column is the generated DC-space with the ACPG and NGA methods. The start position (red star) and the target position (green star) are connected with the red line. The third column is the generated path after projecting the path back to the C-space.

More examples in various ways that the obstacles attach to the boundary are shown in Figure 11. With the implementation of the NGA method, the space distortion is properly handled, and exemplary trajectories are obtained.

### 5.3. Trajectory Optimization

The path planned by the DC-space method is not optimal in C-space as a result of the space distortion. In this section, a trajectory optimization algorithm is proposed to shorten the path L in C-space.

The path L is composed of connected cells after projecting a straight path $L^{\prime }$ in DC-space back to C-space, and is represented as $L=\left\{G_{L}^{1},G_{L}^{2},\cdots ,G_{L}^{l},\cdots ,G_{L}^{n_{l}}\right\}$, where $G_{L}$ is the connected cell in C-space along L and $n_{l}$ is the number of path cells. The optimized trajectory is generated by pruning unnecessary cells based on the collision-free principle, which means if the straight line connecting two cells is not blocked by an obstacle, it is feasible. First, $G_{L}^{1}$ is taken as the temporary start cell, and the adjacent sequence cell $G_{L}^{2}$ is taken as the temporary target cell. Use the collision-free principle to check the feasibility of the straight line $G_{L}^{1}G_{L}^{2}.$ If it is obstacle-free, change the temporary target cell to $G_{L}^{3}$, and if it collides with an obstacle, record $G_{L}^{1}G_{L}^{2}$ as a segment of the optimized path and change the temporary start cell as $G_{L}^{2}$ and repeat the process.

**Figure 12:**
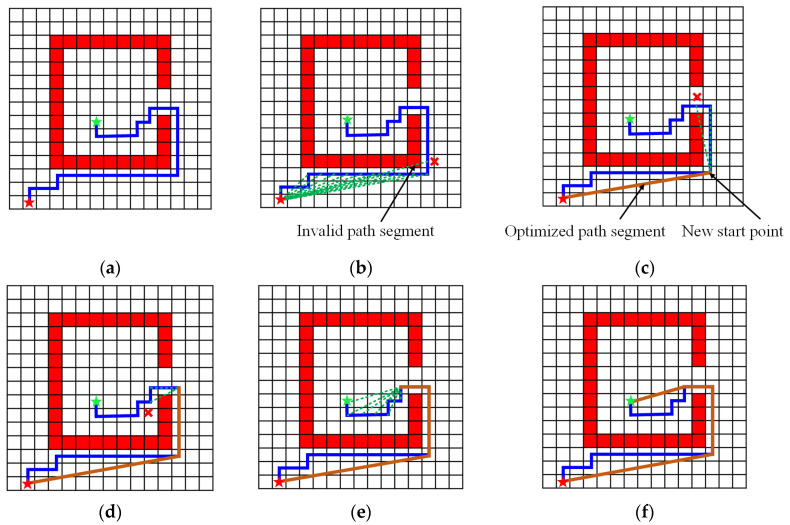
A 2D example of the path optimization process. (**a**) The projected back path (in blue) and the obstacle (in red) in C-space. (**b**) Connecting the start cell (red star) and temporary target cell (green star) until the path conflicts with the obstacle. (**c**) Taking the last cell before the temporary target cell as the new start cell and repeating the step (**b**). (**d**,**e**) Repeating the above two steps until the temporary target cell reaches the final target cell. (**f**) The final optimized trajectory (in orange).

Figure 12 illustrates an example of the optimization process. Figure 12a shows the original path planned by the DC-space method. In Figure 12b, let the first cell to be the temporary start cell and check the following cells as a series of temporary target cells until the path is blocked by the obstacle, which is shown by a red cross marker. Then the last straight line before blocking is taken as a segment of the optimized path, and its ending cell becomes the new start point. The process iterates, as shown in Figure 12c to Figure 12e, and the final optimized path in Figure 12f is obtained when it reaches the target point. Compared with the original blue path, the optimized path is much shorter. The pseudo-code of the trajectory optimization method is given in Algorithm 2.
**Algorithm 2.** Trajectory optimization method.**Input:** original path L, C-space, obstacles $T_{i}$, start point p, target point q**Output:** optimized path $L^{opt}$$L^{opt}=\left[\right]$

$L_{temp}=[]$**for**
l=1 to $n_{l}$
**do**
 **if**
*l* = 1 **then**  $L^{opt}.append\left(G_{L}^{l}\right)$  $start_{temp}=G_{L}^{l}$  continue **end if** $end_{temp}$ = $G_{L}^{l}$ **if**
$l=n_{l}$
**then**  $L^{opt}.append\left(end_{temp}\right)$  **return**
$L^{opt}$ **end if** check whether the path between $start_{temp}$ and $end_{temp}$ intersects with any obstacle $T_{i}$** if** intersection **then**  $L^{opt}.append\left(G_{L}^{l-1}\right)$  $start_{temp}=G_{L}^{l-1}$ **else**  continue **end if****end for**

## 6. Performance Discussion

The proposed ACPG and NGA algorithms provide modifications and complements of the original DC-space method. To clarify the terminology, path planning with ACPG and NGA is called the IDCS method, and the IDCS method with post-optimization is called the Opti-IDCS method in the following content. Although it was sufficiently discussed in [22] that the DC-space method could significantly decrease computation cost compared to classic path planning methods, it is still worth to evaluate the performance of the IDCS method in calculation efficiency and path length with the proposed modifications. The rapidly exploring random tree method, which is widely used in path planning for robot manipulators [27], is used as a comparative study. All the algorithms are implemented in MATLAB [28] and are carried out on a computer equipped with an Intel Core i5-3230M CPU and 12 Gb memory. For each algorithm, it runs ten times for an environment to get the average path generation time and path length. The RRT method can provide largely unlike paths for different runs due to its inherent randomness [29], however, even though the path generation time for IDCS method and Opti-IDCS method vary for each running time, the generated paths are consistent when the environment with obstacles is fixed.

### 6.1. Comparisons of Efficiency and Path Length

In order to evaluate the proposed IDCS and Opti-IDCS methods, the simulations of path planning in ten 2D maps and six 3D maps with various obstacle distributions are carried out. The results are compared with the RRT method.

**Figure 13:**
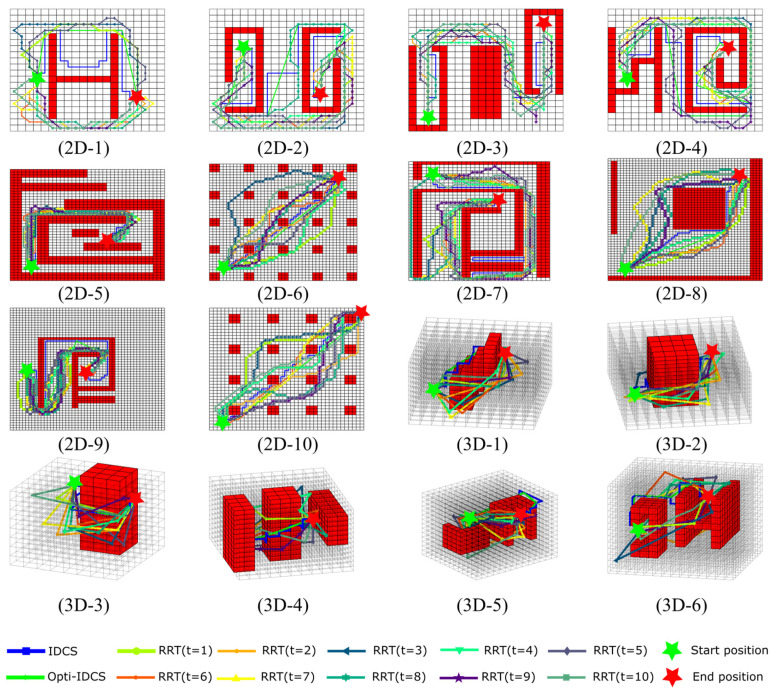
The planned path for various maps. The results generated by the IDCS and Opti-IDCS methods keep the same for each experiment; the path generated by the RRT method is different for each experiment.

Figure 13 shows the generated path for each experiment, and the statistic results for path generation time and path length are summarized in Table 1, Table 2, Table 3 and Table 4. Only one path is illustrated in the figure for IDCS and Opti-IDCS methods since the generated path is invariant for each running time. Although the average path lengths are given in Table 3 and Table 4, it is still unsure how long the path could be and how much the running time it could cost for a particular run. This is an undesirable property for industry applications. Besides, the computational efficiency of RRT highly depends on the map features. For example, though the map size of 2D-6 and 2D-7 are identical, the time cost of the latter is 8.67 times of the former due to its increased complexity. On the other hand, the size of the map also plays an important role in influencing the time cost, which is illustrated by the extremely long time taken for 2D-9. The influence of map size will be further discussed in Section 6.2.

In contrast to RRT, the IDCS method not only decreases the calculation time significantly, but also maintains a stable performance for various maps. In other words, the computation efficiency of the IDCS method shows small sensitivity to the map size and complexity. For instance, the minimum calculation time for the IDCS method over the ten 2D maps is 0.014 s, which is for map 2D-9, and the maximum calculation time is only 0.052 s, which is for map 2D-4. On the contrary, the time cost of the RRT method largely depends on the map situations. For example, it takes 1.289 s for map 2D-4 but 15.076 s for map 2D-9. It is normal for Opti-IDCS method to take more computation time than IDCS method since it is based on the result of IDCS. The Opti-IDCS helps to shorten the path by 22% for 2D maps and 32% for 3D maps on average compared to IDCS. The time-consuming part for Opti-IDCS method is the intersection checking progress during the optimization, but the Opti-IDCS method is still faster than RRT method in 2D and 3D environments. In terms of the path length, usually, the path generated based on IDCS is longer than RRT, which is expected. After a path optimization with Opti-IDCS, the path length is shorter or almost the same as the RRT method. Overall, IDCS and Opti-IDCS methods are more stable and robust than the RRT algorithm. Compared with the IDCS and RRT methods, the Opti-IDCS method can achieve a relatively shorter path without a significant time cost increment.

### 6.2. Map Size vs. Time Cost

It has been shown in Section 6.1 that the map size is related to the time cost for path planning, and this reflects the computation complexity of the algorithms. More rigorous examinations are shown in this section to exclude the influence of map complexity and to study purely on the relationship between the map size and computation time. For this purpose, the same map is discretized with various resolutions, and the time costs of the three algorithms are examined.

**Figure 14:**
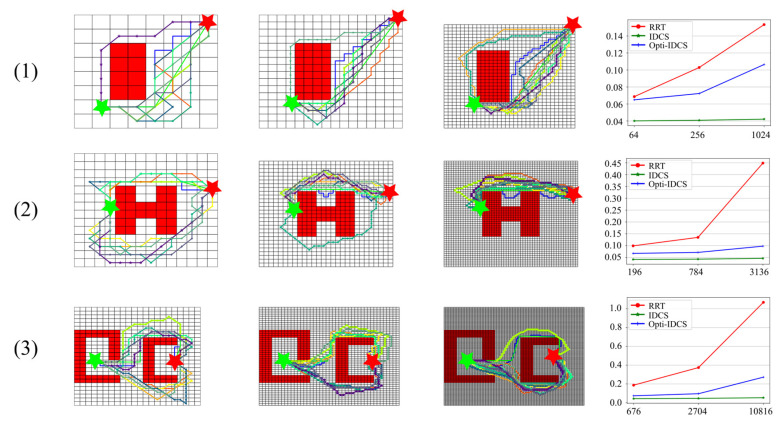

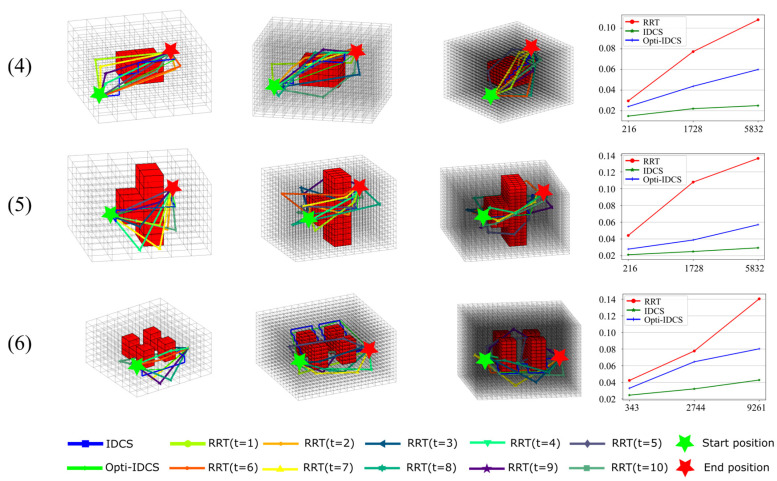
Relationships between the map size and calculation time. The first three columns are the maps with different resolutions, and the fourth column shows the relationship between the number of cells in the map and the time cost.

The results are presented and compared in Figure 14 and Table 5. Six maps (three 2D maps and three 3D maps) are used for evaluation, and each has three different levels of resolutions. For all environments, the path generation time of RRT method rises the most quickly with the increase of the number of map cells. Despite the path generation time for the Opti-IDCS method also grows, it does not perform a sharp trend of increment as RRT. Compared with the RRT method and the Opti-IDCS method, the performance of the IDCS method does not increase obviously in different resolutions of maps, and the computation time is much less than the other two algorithms in the same map, especially in high-resolution maps. Taking experiment 3 as one example, the path generation time is short when the map is in the initial resolution (676 cells), which are 0.187 s, 0.044 s, 0.074 s for RRT method, IDCS method, and the Opti-IDCS method separately. However, when the map size increases to 2704, the path generation time for RRT, IDCS, and the Opti-IDCS methods grows to 0.374 s, 0.047 s, 0.096 s, respectively, which is an almost 100% increment for the RRT method, whereas, the IDCS and Opti-IDCS methods increase about 7% and 30% separately. Moreover, the path generation time for RRT increases to 1.069 s when the map size is 10,816, which is 472% larger comparing to the initial computational time. However, the computational time for IDCS and Opti-IDCS method only increase by 20% (0.053 s) and 268% (0.272 s) compared to the initial computational time. Overall, the average time costs of IDCS are 47%, 29% and 20% of RRT for maps with small, medium and large size in the tests, and the average time costs of Opti-IDCS are 71%, 54% and 45% of RRT in these cases, respectively. Additionally, the average time costs of RRT in medium and large size maps increase 96% and 275% compared to the time in small size maps. As for IDCS and Opti-IDCS methods, the corresponding increments are only 18% and 44% for medium size maps, 35% and 129% for large size maps.

The comparison between experiment 4 and experiment 5 reflects that the shape of the obstacle also influences the path generation time. However, the time cost for IDCS method remains stable even though the obstacles are very different.

## 7. Experiments and Discussion

### 7.1. Platform Introduction

**Figure 15:**
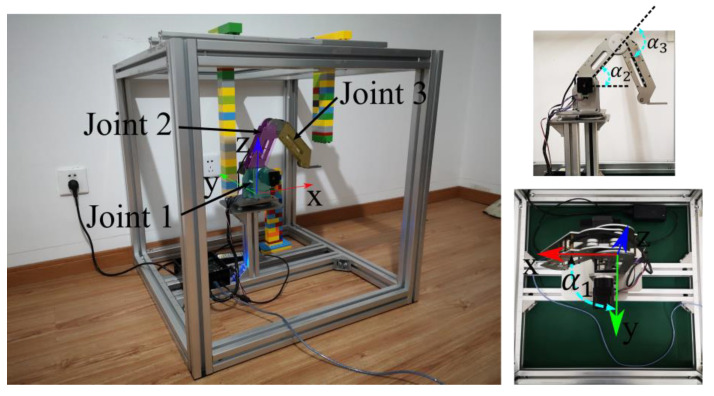
The experimental platform for demonstration purpose.

To demonstrate the feasibility and effectiveness of the algorithm proposed in this paper, an experimental platform is constructed as depicted in Figure 15, which includes a manipulator that sits in a static environment with obstacles. The manipulator is a three DOF DOBOT, which is a multifunctional desktop robotic arm for practical training education. In order to keep the terminal tool horizontal, there is a certain constraint between the second joint and the third joint of the robot, which impacts the construction of its configuration space. The frame size is 600 mm × 600 mm × 750 mm to hold the robot and place obstacles. The obstacles are built and placed in the workspace, and their positions are measured with a tape ruler manually. The robot coordinate system is defined as shown in Figure 15, and the origin is at the center of the robot base.

### 7.2. Experiments and Discussion

**Figure 16:**
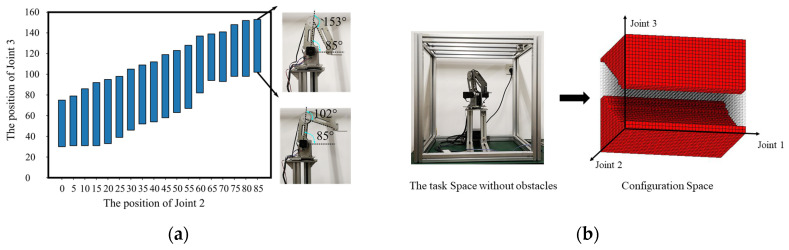
(**a**) The range of Joint 3 changes at different angles of Joint 2. Two extreme positions of Joint 3 are illustrated when the Joint 2 is at 85 degrees. (**b**) The task space without obstacles and the corresponding configuration space.

The experiments are conducted for two different scenarios. The first step is to examine the reachable and unreachable spaces in the C-space, as mentioned in Section 3. The joint angle resolution is set as 5 degree. For DOBOT, Joint 1 can rotate around the base from 0 to 360 degrees. However, the range of Joint 2 is limited to be 0 to 85 degrees, and the range of Joint 3 is constraint by Joint 2, as shown in Figure 16a. Therefore, the corresponding configuration space for the task space without obstacles is given in Figure 16b, where the unreachable space $C_{ur}$ is marked as red blocks.

**Figure 17:**
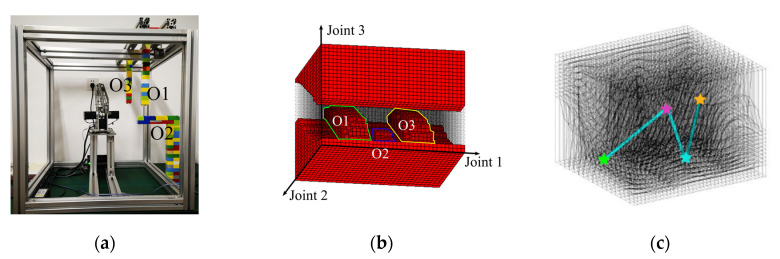
The setup of the first experimental environment, O1, O2, and O3 respond to the corresponding obstacles. (**a**) The setup of the environment. (**b**) The configuration space of the first environment. (**c**) The distorted configuration space. The start point is colored by green, and the target point is colored by yellow. Two passing points are colored by purple and blue, respectively.

**Figure 18:**
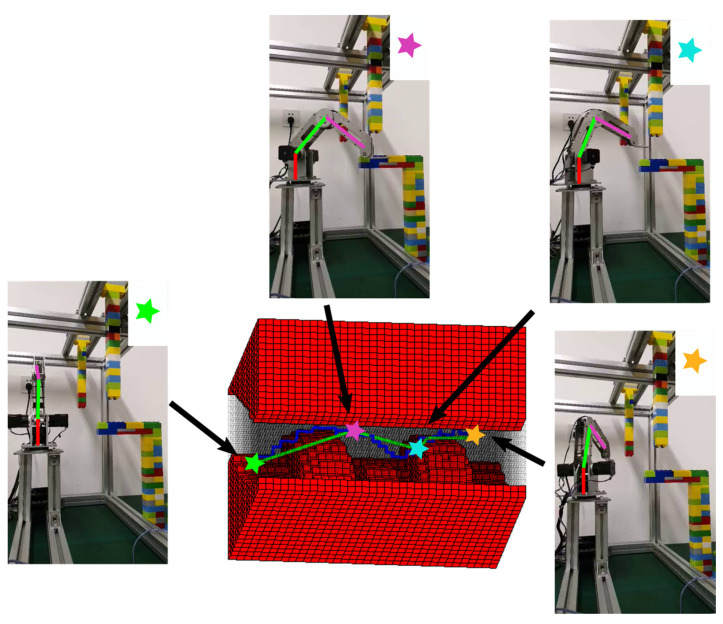
The path generated with the IDCS method (dark blue) and the Opti-IDCS method (green) for the first experiment. The robot poses when it reaches the specified positions are shown as well.

The projection of the obstacle in C-space $C_{ob}$ can be obtained with the method in Section 3. The resulted obstacle map in C-space for the first scenario is shown in Figure 17. The obstacles are placed in the task space as shown in Figure 17a, and the union of corresponding $C_{ob}$ with the $C_{ur}$ in Figure 16b results in the map in Figure 17b. The manipulator is specified to pass through four positions, including the start point, two passing points, and the target point. Their positions in the DC-space are shown in Figure 17c. The resulted paths in the original C-Space by the IDCS method and the Opti-IDCS method, as well as the corresponding robot motion sequences in the physical task space, are shown in Figure 18.

Comparison of the configuration space in Figure 17b and Figure 16b shows the occupied cells caused by the obstacles make the passable area more complicated and narrower. With the known configuration space, the path generation time for IDCS and Opti-IDCS methods are 0.086 s and 0.406 s respectively. Although the passing points are intentionally chosen close to the obstacle, both paths generated by the IDCS method and the Opti-IDCS method can successfully avoid the obstacles and go through the two passing points in experiments. But the latter one is much shorter, and lots of small segments in the path are eliminated. The running tests for both paths demonstrate that the planned path can guarantee obstacle-free operations for the real system. Keeping the running speed of the manipulator be constant, it takes about 120 s for the manipulator to complete the path generated with IDCS method, and only about 25 s for the manipulator to accomplish the path generated by the Opti-IDCS method. Therefore, if the real-time demand is not strong in the task, Opti-IDCS would be the more desirable choice.

**Figure 19:**
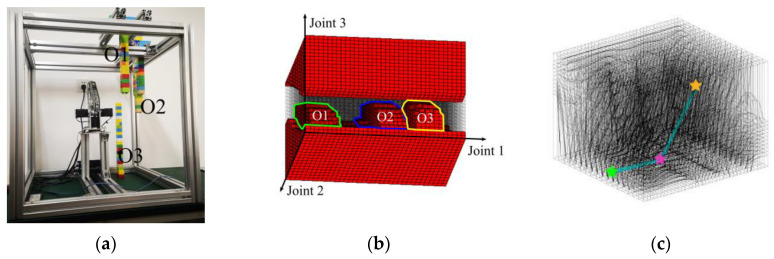
The setup of the second experimental environment. (**a**) The setup of the environment. (**b**) The configuration space of the second environment. (**c**) The distorted configuration space. The start point is colored by green, and the target point is colored by yellow. One passing point is colored by purple.

**Figure 20:**
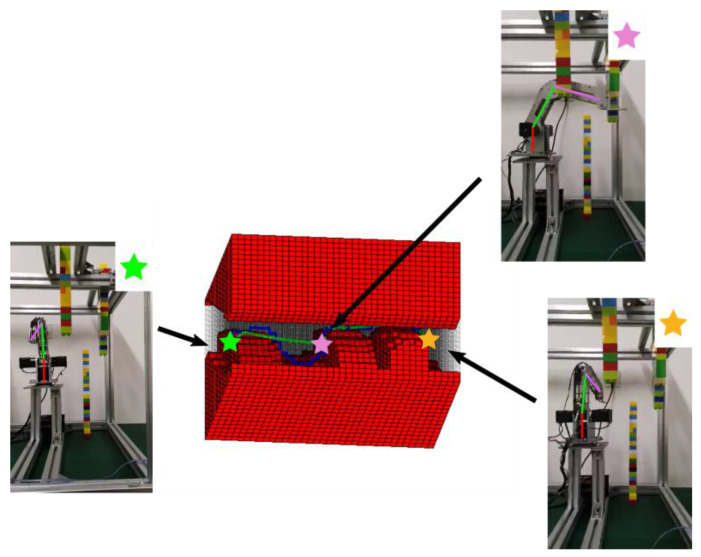
The path generated with the IDCS method (dark blue) and the Opti-IDCS method (green) for the second experiment. The robot pose when it reaches the specified position is shown as well.

We change the positions, shapes, and sizes of the obstacles for the second test and add one passing point in the task space. As shown in Figure 19, the configuration space changed accordingly, and the path planning task seems to be more challenging with very little room to pass through. The two algorithms can still find feasible paths, and the robot fulfilled the mission to move from the start point to the target point and went through the dangerous passing point in the experiment, as shown in Figure 20. Eventually, it takes 0.069 s and 0.460 s for IDCS method and Opti-IDCS method to generate the path, respectively. The manipulator takes about 133 s to accomplish the path generated by the IDCS method and only about 38 s to finish the Opti-IDCS based path. The second test shows that the path planning methods can cope with difficult task setups, and the performances of the two algorithms are similar to test 1. Generally speaking, for tasks requiring fast planning, the IDCS method outperforms the Opti-IDCS method, and for tasks requiring short path and operation time, the Opti-IDCS method provides a superior solution.

## 8. Conclusions and Future Work

Path planning in the distorted configuration space is a novel planning strategy proven to outperform the traditional search-based approaches in computation efficiency. With the central distorted configuration space method preserved, this paper proposed algorithms to solve the problems in the original version by allowing autonomous obstacle collapse, using a node growing algorithm to keep convex boundaries, and optimizing the path with a post-process. These improvements help to solve many practical problems and expand the application scenarios of the original DC-space method.

In addition, computational efficiency analysis is provided to prove that the proposed improvements do not affect the advantages of the DC-space method in computational efficiency. In the analysis of Section 6.2, the average time costs of IDCS are 47%, 29% and 20% of RRT for maps with small, medium and large size in the tests, and the average time costs of Opti-IDCS are 71%, 54% and 45% of RRT, respectively. Besides, the average time increase of RRT in medium and large size maps are 96% and 275% compared to the small size maps, but only 18% and 35% for IDCS, and 44% and 129% for Opti-IDCS. The results not only show the significant improvement of the proposed method on computational efficiency but also reflect the low computational complexity of the algorithm. The path optimization in Opti-IDCS is proven to be effective in decreasing path length by 22% in 2D tests and 32% in 3D tests. Therefore, the users can choose between IDCS and Opti-IDCS according to their task emphasis on computation efficiency or path length. The proposed methods are testified on a 3-DOF manipulator and proven to be capable of solving the obstacle avoidance problem in clustered working environments.

However, the authors have to mention that there is one situation the current IDCS method cannot handle, which are maps with obstacles that are not homeomorphic to a disk or a sphere in high DOF space. Since the obstacles collapse to a point in the DC-space method, which geometrically violates the topology of such obstacles, and the space structure could be destroyed. In this case, neither the original DC-space nor the IDCS in this paper can solve the problem, which remains to be a research topic in the future.

## Figures and Tables

**Table 1:** Path generation time in 2D map (s).

	**1**	**2**	**3**	**4**	**5**
IDCS	0.040 ± 0.004	0.051 ± 0.002	0.050 ± 0.002	0.052 ± 0.002	0.049 ± 0.003
Opti-IDCS	0.070 ± 0.003	0.113 ± 0.005	0.094 ± 0.016	0.123 ± 0.013	0.128 ± 0.005
RRT	0.194 ± 0.036	1.145 ± 0.383	0.824 ± 0.107	1.289 ± 0.233	0.514 ± 0.155
	**6**	**7**	**8**	**9**	**10**
IDCS	0.040 ± 0.002	0.044 ± 0.002	0.043 ± 0.004	0.014 ± 0.003	0.043 ± 0.003
Opti-IDCS	0.115 ± 0.009	0.156 ± 0.006	0.161 ± 0.008	0.106 ± 0.012	0.094 ± 0.004
RRT	0.267 ± 0.060	2.315 ± 0.375	0.301 ± 0.050	15.076 ± 2.297	1.409 ± 0.380

**Table 2:** Path generation time in 3D maps (s).

	1	2	3	4	5	6
IDCS	0.042 ± 0.006	0.046 ± 0.011	0.067 ± 0.006	0.063 ± 0.012	0.071 ± 0.006	0.079 ± 0.010
Opti-IDCS	0.066 ± 0.010	0.071 ± 0.013	0.083 ± 0.008	0.093 ± 0.011	0.157 ± 0.008	0.126 ± 0.016
RRT	0.113 ± 0.021	0.113 ± 0.024	0.086 ± 0.014	0.114 ± 0.015	0.158 ± 0.013	0.170 ± 0.034

**Table 3:** Path length in 2D maps.

	**1**	**2**	**3**	**4**	**5**
IDCS	44 ± 0.0	64 ± 0.0	54 ± 0.0	66 ± 0.0	60 ± 0.0
Opti-IDCS	29.242 ± 0.0	51.835 ± 0.0	46.220 ± 0.0	54.610 ± 0.0	55.835 ± 0.0
RRT	27.762 ± 4.921	52.951 ± 3.401	52.181 ± 3.075	50.858 ± 3.485	60.536 ± 1.738
	**6**	**7**	**8**	**9**	**10**
IDCS	66 ± 0.0	129 ± 0.0	76 ± 0.0	98 ± 0.0	72 ± 0.0
Opti-IDCS	47.045 ± 0.0	97.730 ± 0.0	59.261 ± 0.0	70.673 ± 0.0	51.698 ± 0.0
RRT	55.537 ± 3.651	97.830 ± 15.114	67.917 ± 4.274	75.795 ± 3.185	60.596 ± 3.236

**Table 4:** Path length in 3D maps.

	1	2	3	4	5	6
IDCS	21 ± 0.0	20 ± 0.0	12 ± 0.0	21 ± 0.0	39 ± 0.0	23 ± 0.0
Opti-IDCS	13.555 ± 0.0	13.998 ± 0.0	9.553 ± 0.0	13.303 ± 0.0	24.895 ± 0.0	15.381 ± 0.0
RRT	16.722 ± 1.72	16.728 ± 1.51	13.111 ± 1.76	19.156 ± 2.28	19.220 ± 1.80	18.650 ± 3.93

**Table 5:** Path generation time in maps of different sizes.

	Map Size	IDCS	Opti-IDCS	RRT
Map 1	64	0.040 ± 0.003	0.065 ± 0.004	0.069 ± 0.011
	256	0.041 ± 0.003	0.072 ± 0.003	0.103 ± 0.020
	1024	0.042 ± 0.003	0.106 ± 0.006	0.153 ± 0.023
Map 2	196	0.042 ± 0.002	0.067 ± 0.003	0.099 ± 0.022
	784	0.043 ± 0.001	0.071 ± 0.003	0.135 ± 0.029
	3136	0.046 ± 0.003	0.098 ± 0.004	0.449 ± 0.168
Map 3	676	0.044 ± 0.002	0.074 ± 0.004	0.187 ± 0.061
	2704	0.047 ± 0.005	0.096 ± 0.006	0.374 ± 0.127
	10,816	0.053 ± 0.004	0.272 ± 0.015	1.069 ± 0.464
Map 4	216	0.015 ± 0.003	0.024 ± 0.004	0.030 ± 0.010
	1728	0.022 ± 0.005	0.044 ± 0.008	0.077 ± 0.020
	5832	0.025 ± 0.003	0.060 ± 0.007	0.108 ± 0.031
Map 5	216	0.021 ± 0.005	0.028 ± 0.005	0.044 ± 0.011
	1728	0.025 ± 0.005	0.039 ± 0.007	0.108 ± 0.032
	5832	0.029 ± 0.006	0.057 ± 0.005	0.136 ± 0.024
Map 6	343	0.025 ± 0.005	0.033 ± 0.005	0.042 ± 0.011
	2744	0.032 ± 0.006	0.065 ± 0.005	0.078 ± 0.014
	9261	0.043 ± 0.010	0.080 ± 0.014	0.140 ± 0.048
